# Sedentary Patterns, Physical Activity, and Cardiorespiratory Fitness in Association to Glycemic Control in Type 2 Diabetes Patients

**DOI:** 10.3389/fphys.2017.00262

**Published:** 2017-04-28

**Authors:** Luís B. Sardinha, João P. Magalhães, Diana A. Santos, Pedro B. Júdice

**Affiliations:** Exercise and Health Laboratory, CIPER, Faculdade de Motricidade Humana, Universidade de LisboaCruz-Quebrada, Portugal

**Keywords:** sedentary time, breaks in sedentary time, physical activity, cardiorespiratory fitness, glycemic control, type 2 diabetes

## Abstract

**Background:** Sedentary behavior has been considered an independent risk factor for type-2 diabetes (T2D), with a negative impact on several physiological outcomes, whereas breaks in sedentary time (BST) have been proposed as a viable solution to mitigate some of these effects. However, little is known about the independent associations of sedentary pursuits, physical activity, and cardiorespiratory fitness (CRF) variables with glycemic control. We investigated the independent associations of total sedentary time, BST, moderate-to-vigorous physical activity (MVPA), and CRF with glycemic outcomes in patients with T2D.

**Methods:** Total sedentary time, BST, and MVPA were assessed in 66 participants (29 women) with T2D, using accelerometry. Glucose and insulin were measured during a mixed meal tolerance test, with the respective calculations of HOMA-IR and Matsuda index. Glycated hemoglobin (HbA1c) was also analyzed. CRF was measured in a maximal treadmill test with breath-by-breath gases analysis. Multiple regressions were used for data analysis.

**Results:** Regardless of CRF, total sedentary time was positively associated with HbA1c (β = 0.25, *p* = 0.044). Adjusting for MVPA, total sedentary time was related to fasting glucose (β = 0.32, *p* = 0.037). No associations between total sedentary time and the remaining glycemic outcomes, after adjusting for MVPA. BST had favorable associations with HOMA-IR (β = −0.28, *p* = 0.047) and fasting glucose (β = −0.25, p = 0.046), when adjusted for MVPA, and with HOMA-IR (β = −0.25, *p* = 0.036), Matsuda index (β = 0.26, *p* = 0.036), and fasting glucose (β = −0.22, *p* = 0.038), following adjustment for CRF. When adjusting for total sedentary time, only CRF yielded favorable associations with HOMA-IR (β = −0.29, *p* = 0.039), fasting glucose (β = −0.32, *p* = 0.012), and glucose at 120-min (β = −0.26, *p* = 0.035), and no associations were found for MVPA with none of the metabolic outcomes.

**Conclusion:** The results from this study suggest that sedentary time and patterns are relevant for the glycemic control in patients with T2D. Still, MVPA and CRF counteracted most of the associations for total sedentary time but not for the BST. MVPA was not associated with metabolic outcomes, and CRF lost some of the associations with glycemic indicators when adjusted for total sedentary time. Future interventions aiming to control/improve T2D must consider reducing and breaking up sedentary time as a viable strategy to improve glycemic control.

## Introduction

Global age-standardized diabetes prevalence has increased from 4.3% in 1980 to 9.0% in 2014 in men, and from 5.0 to 7.9% in women, which together with the population growth and aging has led to a near quadrupling of the number of adults with diabetes worldwide (American Diabetes Association, 2016). Prospective studies (Pan et al., 1997; Tuomilehto et al., 2001; Knowler et al., 2002) have shown that moderate-to-vigorous physical activity (MVPA) is associated with a reduction in the risk of type 2 diabetes (T2D). More recently the Look Ahead Multicenter Study concluded that enhancements in MVPA significantly improved the management of cardiovascular diseases risk factors, and thereby reduced the use of medication and expenses associated with T2D treatments (Redmon et al., 2010; Moura et al., 2014). Similarly to the effects of structured exercise, a recent systematic review (Smith et al., 2016) showed that higher leisure time physical activity (PA) was also associated with lower incidence of T2D, and that additional benefits can be achieved if participants engage in considerably higher doses of PA than those suggested by public health recommendations (Smith et al., 2016).

Exercise-stimulated signal transduction can restore glucose metabolism in insulin-resistant muscle through both acute activation of glucose transport and by improving insulin sensitivity for up to 48 h after exercise (Sylow et al., 2016). Increasing PA in adults with T2D has resulted in partial or complete remission of T2D in 11.5% of participants within the first year of intervention and an additional 7% had partial or complete remission of T2D after 4 years of exercise intervention (Gregg et al., 2012). Transgenerational epigenetic research found that acute exercise also leads to transient changes in DNA methylation in adult skeletal muscle (Barres et al., 2012), that may improve glucose homeostasis.

Recently, sedentary behavior has been associated with hyperinsulinemia (Helmerhorst et al., 2009), and increased risk of T2D in the short (Rockette-Wagner et al., 2015) and long term (Hu et al., 2003; Helmerhorst et al., 2009; Grontved and Hu, 2011; Lahjibi et al., 2013), and has also been considered as an independent risk factor for T2D and premature mortality (Grontved and Hu, 2011; van der Ploeg et al., 2012). In a 4-year follow-up, T2D patients who increased sedentary behavior had the greatest increase in waist circumference, independently of MVPA (Lamb et al., 2016). In the short term, sedentary pursuits are related with hyperglycemia (Fritschi et al., 2015), also suggesting acute metabolic effects (Fritschi et al., 2015). Regularization of metabolic control can be achieved by introducing low-intensity physical activity (LIPA), and this can be tracked with an increased mRNA expression of mitochondrial and metabolic genes in skeletal muscle (Osler et al., 2015). However, for individuals presenting a greater imbalance in glycemia, the potential for clinical improvements after these LIPA protocols appears to be limited (Osler et al., 2015).

Preliminary findings indicate that time spent in sedentary behaviors can be reallocated into LIPA or MVPA, with differences in insulin sensitivity, but with greater results for MVPA (Yates et al., 2015b). Replacing sedentary time with LIPA was associated with a 3.0% lower fasting insulin values and a 3.1% lower insulin resistance, using the homeostatic model assessment (HOMA-IR) (Ekblom-Bak et al., 2016). Healy et al. (2011) have previously documented that breaking up sedentary time may be associated with favorable changes in the cardio-metabolic and inflammatory risk profile in adults. These findings have been recently extended to the T2D population, in which interrupting sedentary time by introducing short LIPA breaks may also have the same beneficial effects (Chastin et al., 2015; Duvivier et al., 2016; Dempsey et al., 2016c).

Mounting evidence suggests that breaking up prolonged sedentary time by light ambulation is an effective strategy for improving postprandial glucose regulation (Dunstan et al., 2012; Howard et al., 2013; Latouche et al., 2013; Larsen et al., 2014; Bailey and Locke, 2015; Dempsey et al., 2016a,b), and a recent meta-analysis revealed that breaks of at least light intensity in sedentary periods may have a positive effect on glycemia, independently of total sedentary time (Chastin et al., 2015). Dunstan et al. (2012) found that introducing light walking breaks every 20 min (2-min breaks) reduced 5 h glucose incremental area under the curve (iAUC) by 24% and 5 h insulin iAUC by 23%. From this same experiment, interrupting sedentary behavior reduced blood pressure (Larsen et al., 2014), attenuated the increase in plasma fibrinogen (Howard et al., 2013), and it also induced changes in the expression of skeletal muscle genes involved in cellular development, growth and proliferation, and lipid and CHO metabolism in non-diabetic adults (Latouche et al., 2013). Another study with a similar experimental approach also found that light walking reduced 5 h blood glucose iAUC by 15.9% compared to prolonged sitting in healthy individuals (Bailey and Locke, 2015), and that interrupting sedentary time by standing-up did not improve glucose tolerance (Bailey and Locke, 2015). Introducing light walking breaks reduced T2D patients' 7 h glucose, insulin, and C-peptide iAUC, compared with prolonged sitting (Dempsey et al., 2016b). Interestingly, 22 h hyperglycemia was also reduced and glycemic improvements persisted nocturnally, until the following morning (Dempsey et al., 2016a).

Experimental evidence is paramount to establish causal relationships, but the controlled conditions and sometimes unrealistic protocols makes it difficult for an ecological transfer to the real-life settings. Understanding if the associations between breaks in sedentary time (BST) and metabolic indicators remain while in free-living conditions is still unknown. Moreover, patients with lower fitness and high fasting glucose levels benefited more from replacing the same amount of sedentary time with LIPA and MVPA, compared with participants with normal to high cardiorespiratory fitness levels (CRF) (Ekblom-Bak et al., 2016). Additionally, CRF seems to be positively associated with glycemic control (Rohling et al., 2016), and may be an important mediator in the relationship between sedentary behavior and MVPA with metabolic outcomes (Rohling et al., 2016).

Notably, the acute experimental findings have mainly resulted from healthy and overweight participants, and the results seem to be less consistent for T2D patients, with one study showing no association between the number of BST with insulin levels or HOMA-IR (Cooper et al., 2012). Thus, the aim of this study was to cross-sectionally analyze the independent associations for total sedentary time, BST, and MVPA, with fasting glucose, glucose tolerance at 120 min, HOMA-IR and Matsuda index, and glycated hemoglobin (HbA1c), in free-living conditions, and examine if CRF may counteract these associations in T2D patients.

## Materials and methods

### Study design and participants

Sample recruitment was carried out by media, e-mails, or community events. For this cross-sectional study, a total of 96 participants were recruited but, given that 30 participants have not completed all the assessments, the results are based on the 66 participants (29 women) from which we have complete data (accelerometer, blood sample collection, and CRF assessment). In order to be included in this investigation, the participants had to be adults previously diagnosed with T2D in accordance with the ADA criteria (American Diabetes Association, 2016). This study was carried out in accordance with the recommendations of the Declaration of Helsinki for Human Studies (World Medical Association, 2008). The protocol was approved by the Ethics Committee of the Portuguese Diabetes Association (approval number: 07/17/2013). Written informed consent was obtained from all participants before entering the study and prior to any protocol-specific procedures.

### Anthropometry and body composition

Participants were weighed to the nearest 0.01 kg while wearing minimal clothes and without shoes, on an electronic scale (Seca, Hamburg, Germany). Height was measured to the nearest 0.1 cm with a stadiometer (Seca, Hamburg, Germany) according to the standardized procedures described elsewhere (Lohman et al., 1988). Body mass index (BMI) was calculated as body mass (kg)/height^2^ (m). BMI was further categorized into normal (<25 kg/m^2^), overweight (25–24.9 kg/m^2^), and obese (≥30 kg/m^2^).

Waist circumference measurement was taken with the participant in a standing position, over the naked skin, to the nearest 0.1 cm. The tape was applied horizontally just above the uppermost lateral border of the right ilium at the end of normal expiration (CDC, 2016). The mean of two measurements was considered. If the two measurements differed by more than 1 cm, a third measurement was necessary, and the two closest measurements were averaged. Dual energy X-ray absorptiometry (Hologic Explorer-W, fan-beam densitometer, software QDR for windows version 12.4, Waltham, USA) was used to estimate total body fat. A whole-body scan was performed and the attenuation of X-rays pulsed between 70 and 140 kV synchronously with the line frequency for each pixel of the scanned image was measured. The same laboratory technician positioned the subjects, performed the scans and executed the analyses according to the operator's manual using the standard analysis protocol (Santos et al., 2013). Based on ten participants, the coefficient of variation (CV) in our laboratory for fat mass was 1.7%.

### Objective measures of sedentary time and physical activity

Sedentary time and PA were assessed by accelerometry (ActiGraph, GT3X+ model, Fort Walton Beach, FL, USA). The accelerometer is a small device that measures the acceleration of normal human movements, ignoring high-frequency vibrations associated with mechanical equipment. All participants were asked to wear the accelerometer on the right hip, close to the iliac crest. The device activation, download, and processing were performed using the software Actilife (v.6.9.1) (ActiGraph, Fort Walton Beach, FL, USA). The devices were activated on the first day in the morning and data were recorded using the raw mode with a 100 Hz frequency, and posteriorly downloaded into 15-s epochs. Apart from accelerometer non-wear time (i.e., when it was removed during sleep and water activities), periods of at least 60 consecutive minutes of zero activity intensity counts were also considered as non-wear time. A valid day was defined as having 600 min (10 h) or more of monitor wear, and all participants with at least three valid days (including 1 weekend day) were included in the analyses. Each minute during which the accelerometer counts were below 100 cpm was defined as sedentary time. A break in sedentary time was defined as all interruptions (lasting at least 1-min) in sedentary time when the recorded counts value were >100 cpm. BST were divided by total sedentary time and the variable hourly breaks in sedentary time (BST/ST) was used in the analysis. Accelerometer counts ≥100 cpm were classified as PA with additional separation into light-intensity (LIPA: 100–2,019 cpm) and moderate-to-vigorous intensity (MVPA ≥ 2,020 cpm) (Troiano et al., 2008; Colley et al., 2010). There are no cutoffs for the sedentary time using the three-axial information from this new generation Actigraph GT3X+ accelerometer; therefore we used the previous cutoffs which are based on the vertical-axis only. Compliance with PA recommendations for public health was assessed according to the WHO recommendations (Adults: 150 min/week of MVPA defined as ≥21.4 min/day).

### Cardiorespiratory fitness

Cardiorespiratory fitness was determined using a Bruce standard protocol (Bruce, 1971) on a motorized treadmill to exhaustion (model Q-65, Quinton, Cardiac Science Corp; Bothell, WA, USA). All graded exercise tests were monitored using a 12 lead electrocardiogram PC-based acquisition module (model Quark C12, Cosmed, Rome, Italy) and all data, including heart rate, were monitored and recorded using Cosmed software (Cosmed, Rome, Italy. Inspired and expired gases were continuously analyzed, breath-by-breath, through a portable gas analyzer (K4b2, Cosmed, Rome, Italy). Participants exercised until at least two of the following test termination criteria were reached: (1) participants volitional fatigue; (2) respiratory exchange ratio reached 1.1 or higher; (3) participants reached predicted maximal heart rate; (4) oxygen uptake did not increase in spite of increasing workload. Plateau in oxygen consumption with an increase in workload. The highest 20-s value for oxygen consumption (ml/kg/min) attained in the last minute was used in the analysis.

### Laboratory measurements

After the recruitment process, participants underwent biochemical assessments, including a mixed meal tolerance test and analysis of the HbA1c. Blood samples were collected from an indwelling catheter for the assessment of glucose, insulin, and HbA1c before ingesting the meal, and 30, and 120 min after the beginning of the meal consumption (2 bottles of Boost Complete Nutritional Drink), for glucose and insulin. Samples were drawn into chilled, heparinized tubes and centrifuged rapidly to avoid glycolysis. Plasma glucose was measured by photometry (auto analyzer Olympus AU640, Beckman Coulter). Plasma insulin was analyzed using electrochemiluminescence immunoassays (Liaison, Diasorin). HbA1c was analyzed by immunoassay (auto analyzer Hb9210 Premier A. Menarini diagnostics). Homeostasis model assessments of insulin resistance (HOMA-IR) and the Matsuda index were calculated (Matthews et al., 1985; Matsuda and DeFronzo, 1999) using their respective formulas.

### Statistical analysis

Data analyses were performed using IBM SPSS Statistics version 22.0 (SPSS Inc., an IBM Company, Chicago, Illinois, USA). Descriptive statistics including means ± SD were calculated for all outcome variables. Normality was tested using Q-Q plots. Comparisons between sexes were performed using independent sample *T*-test or the non-parametric Mann-Whitney-Wilcoxon approach.

Multiple regression analyses were performed to understand the associations between total sedentary time, breaks in sedentary time, MVPA (linear and dichotomized as compliance with PA guidelines), and CRF with metabolic variables (HOMA-IR, Matsuda index, HbA1c, fasting glucose, glucose at 120 min). Model adjustments included age, sex, time with diagnosed diabetes, and wear time of the accelerometer. To analyze the independent effects, additional adjustments were performed to for MVPA, CRF, or sedentary time (except when exposure).

During model development, normality and homoscedasticity of residuals were tested. If normality was rejected during model development, a logarithmic function of the dependent variable was used. If more than one variable was a predictor in the model, a variance inflation factor for each independent variable was calculated to evaluate multicollinearity, and values bellow 5 were considered not to have multicollinearity issues (Montgomery and Peck, 1982). For all tests statistical significance was set at *p* < 0.05.

## Results

Descriptive characteristics of the participants are presented in Table 1, for both sexes and for the overall sample.

**Table 1:** **Participants' characteristics, according to sex and all sample**.

	**All Sample (*n* = 66)**	**Women (*n* = 29)**	**Men (*n* = 37)**	***p*-Value**
Age (years)	58.9 ± 8.2	58.7 ± 7.8	59.0 ± 8.6	0.914
Time of Diabetes Diagnosis (years)	7.2 ± 5.0	6.8 ± 4.9	7.6 ± 5.2	0.516
Height (cm)	164.7 ± 8.8	157.2 ± 5.9	170.8 ± 5.5	<0.001^*^
Weight (kg)	83.5 ± 15.6	77.6 ± 12.8	88.2 ± 16.2	<0.001^*^
Body Mass Index (kg/m^2^)	30.8 ± 5.2	31.5 ± 5.2	30.2 ± 5.1	0.335
Waist Circumference (cm)	103.5 ± 12.5	102.0 ± 11.5	104.6 ± 13.4	0.425
Percentage Body Fat (%)	34.4 ± 7.1	40.7 ± 3.1	29.4 ± 5.2	<0.001^*^
Cardiorespiratory Fitness (VO_2_, ml/kg/min)	25.8 ± 5.5	23.4 ± 3.5	27.6 ± 6.1	0.002
Fasting Glucose (mg/dl)	159.6 ± 56.4	153.5 ± 50.9	164.3 ± 60.7	0.486
Glucose 120 min (mg/dl)	272.2 ± 124.5	233.1 ± 91.9	303.7 ± 138.9	0.022^*^
Fasting Insulin (Ul/l)	12.9 ± 8.3	13.8 ± 7.6	12.2 ± 8.9	0.451
HbA1c (%)	7.1 ± 1.3	7.1 ± 1.2	7.2 ± 1.4	0.919
HOMA-IR	5.2 ± 4.3	5.6 ± 4.3	4.9 ± 4.3	0.289
Matsuda Index	4.6 ± 6.5	4.3 ± 7.1	4.8 ± 6.3	0.482
Total Sedentary Time (min/day)	582.3 ± 79.8	575.8 ± 71.4	587.5 ± 86.7	0.573
Breaks in Sedentary Time per Sedentary Hour (number/h)	7.8 ± 3.8	8.6 ± 3.6	7.2 ± 3.9	0.055
Light Physical Activity (min/day)	214.9 ± 71.0	241.5 ± 66.5	193.8 ± 68.1	0.005^*^
Moderate-to-Vigorous Physical Activity (min/day)	33.7 ± 24.4	26.4 ± 16.9	39.5 ± 27.9	0.072

*HbA1c, glycated hemoglobin; HOMA-IR, homeostatic model assessment; VO_2_, oxygen consumption*.

* *Significant differences between sexes*.

Overall, 51.5% of the sample was categorized as obese, 31.8% as overweight, and 16.7% as normal weight. No differences were found for age, time of diagnosed diabetes, BMI, and waist circumference, between men and women. CRF (*p* = 0.002) was higher in men when compared to women, whereas percentage body fat (*p* < 0.001) was higher in women compared to men. Regarding metabolic and inflammatory variables, with the exception of glucose at 120 min (*p* = 0.022) where males presented higher values, there were no differences between both sexes. Compared to men, women spent a higher amount of time per day in light PA (*p* = 0.005). There were no differences between men and women for sedentary time, breaks in sedentary time per sedentary hour, and time spent engaging in MVPA.

In the multicollinearity diagnosis, we found no variation inflation factor above 5, which is the rule of thumb used in regression models to assess if the β is affected.

Associations for total sedentary time and breaks in sedentary time with metabolic variables are presented in Table 2.

**Table 2:** **Multiple regression analyses for total sedentary time and breaks in sedentary time with metabolic variables**.

	**Model 1^a^**		**Model 2^a^^,^^b^**		**Model 3^a^^,^^c^**	
	**β (CI 95%)**	***p-value***	**β (CI 95%)**	***p-value***	**β (CI 95%)**	***p-value***
**HOMA-IR**						
Sedentary Time	0.30 (0.04;0.55)	0.023^*^	0.33 (−0.001;0.35)	0.051	0.25 (−0.01;0.49)	0.058
BST-ST	−0.28 (−0.51;−0.05)	0.020^*^	−0.28 (−0.54;−0.01)	0.046^*^	−0.25 (−0.47;−0.02)	0.036^*^
**MATSUDA INDEX**						
Sedentary Time	−0.33 (−0.58;−0.05)	0.020^*^	−0.32 (−0.64;0.02)	0.065	−0.26 (−0.52;0.01)	0.058
BST-ST	0.30 (0.05;0.53)	0.017^*^	0.27 (−0.03;0.53)	0.052	0.26 (0.02;0.48)	0.036^*^
**HbA1c**						
Sedentary Time	0.28 (0.04;0.54)	0.022^*^	0.18 (−0.12;0.48)	0.237	0.25 (0.01;0.49)	0.044^*^
BST-ST	−0.23 (−0.45;−0.01)	0.038^*^	−0.15 (−0.40;0.10)	0.240	−0.21 (−0.43;0.01)	0.059
**FASTING GLUCOSE**						
Sedentary Time	0.29 (0.05;0.52)	0.018^*^	0.32 (0.02;0.62)	0.037^*^	0.23 (−0.002;0.45)	0.052
BST-ST	−0.26 (−0.47;−0.04)	0.021^*^	−0.25 (−0.50;-0.004)	0.047^*^	−0.22 (−0.43;−0.01)	0.038^*^
**GLUCOSE 120 MIN**						
Sedentary Time	0.29 (0.05;0.52)	0.020^*^	0.14 (−0.16;0.43)	0.362	0.24 (−0.001;0.47)	0.051
BST-ST	−0.20 (−0.42;0.02)	0.075	−0.08 (−0.32;0.17)	0.530	−0.17 (−0.38; 0.05)	0.124

β, standardized beta coefficient; CI, confident interval; HOMA-IR, homeostatic model assessment; BST-ST, breaks in sedentary time per sedentary hour; HbA1c, glycated hemoglobin.

* *Significant at p < 0.05*.

a *Adjusted for age, sex, time with diagnosed diabetes, and wear time of the accelerometer*.

b *Adjusted for moderate-to-vigorous physical activity*.

c *Adjusted for cardiorespiratory fitness*.

Following adjustment for covariates, including age, sex, time of diabetes diagnosis, and wear time (Table 2, model 1), detrimental linear associations for total sedentary time with all glycemic outcomes were found. Conversely, except for glucose measured at 120 min, favorable associations were found for the breaks in sedentary time with all the metabolic variables.

Following an additional adjustment for time spent in MVPA (Table 2, model 2), total sedentary time yielded a detrimental association with fasting glucose (β = 0.32, *p* = 0.037), whereas, breaks in sedentary time remained favorably associated with HOMA-IR (β = −0.28, *p* = 0.047) and fasting glucose (β = −0.25, *p* = 0.046). The remaining metabolic outcomes were no longer associated with both total sedentary time and breaks in sedentary time after adjusting for MVPA.

In the last model (Table 2, model 3), adjusted for both the covariates of model 1 and CRF, total sedentary time remained detrimentally associated with HbA1c (β = 0.25, *p* = 0.044), while breaks in sedentary time had favorable associations with HOMA-IR (β = −0.25, *p* = 0.036), Matsuda index (β = 0.26, *p* = 0.036), and fasting glucose (β = −0.22, *p* = 0.038). Similarly to model 2, total sedentary time and breaks in sedentary time were no longer associated with all the remaining metabolic variables, after adjusting for CRF.

Table 3 reports the standardized coefficients for MVPA and CRF with metabolic outcomes. Overall, MVPA was negatively associated with glucose at 120 min (β = −0.31, *p* = 0.006) and with HbA1c (β = −0.26, *p* = 0.026), whereas, CRF had favorable associations with HOMA-IR (β = −0.34, *p* = 0.016), Matsuda index (β = 0.32, *p* = 0.025), fasting glucose (β = −0.36, *p* = 0.004), and glucose at 120 min (β = −0.31, *p* = 0.014). When adjusting for total sedentary time, only CRF yielded favorable associations with HOMA-IR (β = −0.29, *p* = 0.039), fasting glucose (β = −0.32, *p* = 0.012), and glucose at 120 min (β = −0.26, *p* = 0.035), and no associations were found for the remaining metabolic outcomes.

**Table 3:** **Multiple regression analyses for moderate-to-vigorous physical activity and cardiorespiratory fitness with metabolic variables**.

	**Model 1^a^**		**Model 2^a^^,^^b^**	
	**β (CI 95%)**	***p-value***	**β (CI 95%)**	***p-value***
**HOMA-IR**				
MVPA	−0.15 (−0.40;0.10)	0.241	0.04 (−0.27;0.35)	0.785
CRF	−0.34 (−0.67;−0.07)	0.016^*^	−0.29 (−0.62;-0.02)	0.039^*^
**MATSUDA INDEX**				
MVPA	0.18 (−0.08; 0.44)	0.162	0.01 (−0.31;0.32)	0.971
CRF	0.32 (0.05;0.66)	0.025^*^	0.26 (−0.03; 0.59)	0.075
**HbA1c**				
MVPA	−0.26 (−0.49;−0.03)	0.026^*^	−0.15 (−0.44; 0.13)	0.290
CRF	−0.22 (−0.53;0.04)	0.096	−0.17 (−0.47;0.10)	0.200
**FASTING GLUCOSE**				
MVPA	−0.14 (−0.37;0.10)	0.246	0.37 (−0.23;0.34)	0.716
CRF	−0.36 (−0.68;−0.14)	0.004^*^	−0.32 (−0.63;−0.08)	0.012^*^
**GLUCOSE 120 MIN**				
MVPA	−0.31 (−0.55;−0.10)	0.006^*^	−0.24 (−0.53;0.04)	0.088
CRF	−0.31 (−0.63;−0.07)	0.014^*^	−0.26 (−0.57;−0.02)	0.035^*^

*β, standardized beta coefficient; CI, confident interval; HOMA-IR, homeostatic model assessment; MVPA, moderate-to-vigorous physical activity; CRF, cardiorespiratory fitness; HbA1c, glycated hemoglobin*.

* *Significant at p < 0.05*.

a *Adjusted for age, sex, time with diagnosed diabetes, and wear time of the accelerometer*.

b *Adjusted for total sedentary time*.

Further analyses were conducted to analyze if complying with PA guidelines for MVPA was associated with the metabolic outcomes. In these analyses we verified that not performing at least 150 min of MVPA per week (average 24.1 min/day of MVPA) was associated with Matsuda index (β = −0.36, *p* = 0.005) and with HOMA-IR (β = 0.32, *p* = 0.011), but not with fasting glucose (β = 0.08, *p* = 0.490), HbA1c (β = 0.167, *p* = 0.152), nor glucose at 120 min (β = 0.16, *p* = 0.174). After additional adjustment for total sedentary time, only the association between meeting PA guidelines with Matsuda index remained significant (β = −0.29, *p* = 0.043).

## Discussion

The main findings from this study suggest that total time spent in sedentary activities and the patterns of accumulation are detrimental to the metabolic health of T2D patients. Still, MVPA seemed to offset the associations for both total sedentary time and BST with all metabolic outcomes, except for BST with fasting glucose and HOMA-IR. CRF only counteracted the associations for total sedentary time, whereas the associations for BST with most of the main outcomes remained unaltered. Our results suggest that future interventions aimed to control/improve T2D must consider BST as a viable strategy to improve glycemic control.

Both total MVPA and sedentary time have been consistently associated with HOMA-IR and Matsuda index, but after adjustment for each other, only associations with Matsuda index remained (Yates et al., 2015a). In the current investigation it was verified that, when considering MVPA as a continuous variable, only associations with HbA1c were verified. Interestingly, when considering compliance with PA guidelines as the independent variable, associations were observed for the Matsuda index and HOMA-IR. This finding suggests that there may be a threshold (i.e., 150 min of MVPA per week) to experience the metabolic benefits that are related to MVPA, and therefore more minutes of PA at these intensities do not necessarily relate to glycemic control. From the different variables used to assess glycemic control, both Matsuda index and HOMA-IR have been shown to correlate well with euglycemic-hyperinsulinemic clamp on cross-sectional level, making them suitable insulin resistance surrogates (Lorenzo et al., 2010). Observational evidence suggests that a 30-min difference in total sedentary time was inversely associated with a 4% difference in Matsuda index, whereas every 30 min in MVPA was positively associated with a 13% difference (Yates et al., 2015a). Reallocating 30 min of sedentary time into MVPA was associated with a 15% difference in HOMA-IR and an 18% difference in Matsuda index (Yates et al., 2015b). Our findings suggest that when considering these insulin resistance indexes, no associations remained when adjusting total sedentary time for MVPA and vice-versa.

Baseline MVPA has been documented as a predictor of fasting insulin at follow-up, with a borderline significance for HOMA-IR, regardless of total sedentary time (Ekelund et al., 2009). In contrast, each additional daily hour spent sedentary was cross-sectional associated with a 3% higher fasting insulin and HOMA-IR, but did not predict 5-year changes in metabolic parameters or incidence of metabolic disorders (Barone Gibbs et al., 2015). Experimental data has previously suggested that performing 45 min of MVPA following more than 10 h of sitting had beneficial effects on glucose metabolism in T2D patients (van Dijk et al., 2013), thus, some of the contradicting results may be explained by the specific window of time that both sedentary time and MVPA have in their ability to alter these specific metabolic indicators. Similar to the results observed for mortality, in a harmonized meta-analysis involving more than 1 million men and women (Ekelund et al., 2016), we found in our sample of T2D that adjusting for MVPA eliminated almost all the associations for total sedentary time with glycemic indicators, except fasting glucose. The results for the fasting glucose are in accordance with the findings from a previous systematic review (Brocklebank et al., 2015), and a longitudinal analysis that found higher baseline sedentary time to be associated with 3-year increases in fasting glucose, fasting insulin and HOMA-IR, regardless of MVPA (Lahjibi et al., 2013).

A new finding from the present investigation with T2D patients was that, the associations for BST with HOMA-IR and fasting glucose were not affected by the adjustment for MVPA. These findings further highlight the important role of breaking up sedentary time to improve cardiometabolic markers in the general population (Healy et al., 2011) and in T2D patients using an isotemporal substitution modeling approach (Healy et al., 2011; Falconer et al., 2015), and therefore to encourage adults with diagnosed T2D to adopt BST as a strategy for improving metabolic health. The underlying mechanisms explaining the associations between BST and glycemic control are still relatively unknown, but acute light exercise bouts may activate alternative molecular signals that can bypass defects in insulin signaling in skeletal muscle, resulting in an insulin-independent increase in glucose uptake (Stanford and Goodyear, 2014) through several signal transduction pathways (Sylow et al., 2016), including the AMPK signaling network (Kjobsted et al., 2017), a function that remains intact in T2D patients (Kjobsted et al., 2016). It is important to highlight that the AMPK signaling is intensity-dependent (Birk and Wojtaszewski, 2006), however, it may also be stimulated by an increased energy expenditure resulting from skeletal muscle contractions. Breaking up sedentary time may have benefits that go beyond the physiological mechanisms, including certain energetic changes (i.e., increasing energy expenditure 35% above sitting, and 28% compared to standing while motionless) (Judice et al., 2016), that can justify why BST (frequent muscle contractions throughout the day) were favorably associated with glycemic outcomes in the present study.

Nonetheless, breaking up sedentary time was not independent of MVPA for some metabolic outcomes, particularly the Matsuda index, and HbA1c, which is in line with the results reported by some investigations (Cooper et al., 2012; van der Berg et al., 2016). For example, in a study with 528 adults with newly diagnosed T2D, no associations were found between BST and insulin levels or HOMA-IR (Cooper et al., 2012). Similar results were found in the Maastricht Study with 2497 participants, where an extra hour of sedentary time was associated with increased odds for T2D (22%), but the pattern of sedentary time accumulation was weakly associated with the incidence of metabolic impairment (van der Berg et al., 2016). With different results, Healy et al. (2011) found that, regardless of total sedentary time and MVPA, increased BST were beneficially associated with plasma glucose at 120 min. Additionally, an investigation based on 4935 adults found that total sedentary time was associated with higher insulin, and each additional 10 breaks/day were related to 0.57% lower glucose, and 4.19% lower insulin (Carson et al., 2014).

When considering experimental evidence (Dunstan et al., 2012; Howard et al., 2013; Latouche et al., 2013; Larsen et al., 2014; Bailey and Locke, 2015; Dempsey et al., 2016a,b), breaking up prolonged sedentary time with light ambulation is still an effective strategy for improving glucose regulation, which further clarifies the need to expand current diabetes-related PA guidelines, by introducing regular breaks in prolonged sedentary time (Dempsey et al., 2016c). Dunstan et al. (2012) found that breaking up sedentary time with LIPA bouts reduced 5 h glucose iAUC by 24% and 5 h insulin iAUC by 23%. When considering T2D patients, introducing light walking breaks reduced 7 h glucose, insulin, and C-peptide, compared with prolonged sitting (Dempsey et al., 2016b). The same authors verified that the glycemic improvements that arise from breaking up sedentary time persist until the next morning, indicating that there may be medium to long term benefits in T2D patients (Dempsey et al., 2016a). Even though these experimental findings are of great importance (because they allow establishing causal relationships between BST and metabolic outcomes) the laboratorial settings and protocols in which they are performed, do not mimic real-life conditions and limit their ecological transfer. On the other hand, the presented investigation collected free-living accelerometry data that may reflect a more realistic PA and sedentary pattern profile.

Breaking up sedentary time seems to reverse the effects of chronic inactivity on the expression of some specific genes and molecular processes (Latouche et al., 2013), but some of the contradicting findings for the independent associations of sedentary patterns with glycemic indicators may be explained by CRF, which is usually not accounted for most of the models. CRF is a reliable metric to assess the ability of the cardiovascular system to sustain prolonged physical work, and has been shown to be one the most powerful predictors of mortality and morbidity (Despres, 2016). Poor CRF is an independent risk factor for cardiovascular diseases and related mortality (Despres, 2016), and it appears to be a link between changes in CRF and glycemic control (Larose et al., 2011; Sui et al., 2012; Dickie et al., 2016). Alongside with these results, replacing 30 min of sedentary time with LIPA provided higher benefits in metabolic profile in participants with lower CRF when compared with those with normal to high CRF levels (Ekblom-Bak et al., 2016), suggesting that the associations between sedentary pursuits and metabolic outcomes may be moderated by CRF. There is a lack of studies that analyzed the associations for sedentary time and respective patterns with glycemic indicators while adjusting for CRF (Ekblom-Bak et al., 2016; Rohling et al., 2016). In the present study, after adjusting for CRF, it was observed that total sedentary time was only associated with HbA1c, whereas BST had favorable associations with HOMA-IR, Matsuda index, and fasting glucose. Thus, as previously shown (Ekblom-Bak et al., 2016; Rohling et al., 2016), CRF can neutralize most of the associations for total sedentary time with glycemic outcomes, and this may be explained by the association between total sedentary time and CRF itself (Krogh-Madsen et al., 2010).

A sedentary lifestyle is usually associated with poor levels of CRF (Lakka et al., 2003), but the fact that the associations for BST with HOMA-IR, Matsuda index, and fasting glucose remained independent of CRF, is another novel finding and suggests that BST may not be as influenced by CRF as total sedentary time. To the authors' knowledge, there is no evidence on the associations for BST with CRF in T2D patients, making it necessary to further investigate the plausible mechanisms that underlie these findings. CRF was not associated with all metabolic outcomes, after adjustment for total sedentary time, contradicting previous findings on the independent associations for CRF with metabolic outcomes (Larose et al., 2011; Dickie et al., 2016). Sedentary behavior accumulating pattern is a relatively new research topic and these contradicting findings in the literature reinforce the need for further experimental investigations that may help to uncover this subject.

Regardless of the amount of observational and experimental studies showing the deleterious effects of prolonged sedentary time and the benefits associated with breaking up sedentary time, few studies have focused on T2D patients and none controlled for their CRF levels. These were major strengths of the present study, and one must cautiously account for CRF when examining the associations of PA/sedentary variables with metabolic outcomes in T2D patients, as this covariate may explain some of the variability found in previous investigations (Bouchard et al., 2015). Another important message is that the relative role of total sedentary time, BST, MVPA, and CRF may depend on the glycemic indicators that are being considered, and interpretation must be careful when considering different outcomes in patients with T2D. The present investigation is not without limitations, the inability to establish causality due to the cross-sectional nature of the data is by far the major problem. However, this study provides a basis for future interventional studies to confirm our findings in T2D patients.

## Conclusions

The results from this study suggest that sedentary time and its patterns can be relevant for the glycemic control in patients with T2D. Current international recommendations include 150 min of moderate-intensity activity, or 75 min of vigorous-intensity activity, or some combination of moderate and vigorous activity with at least 2-days of resistance exercise. Thus, the present findings suggest that it will be equally important for T2D prevention and management programs to broaden the focus of public health message, and not only target MVPA, but also endorse people to reduce and interrupt sedentary time more often and improve CRF. Future interventions aiming to control/improve T2D must target reductions in sedentary behavior and increase the number of breaks in sedentary time as a viable strategy to improve glycemic control.

## Author contributions

LBS contributed to the conception and design of the study. JM, DS, and PJ were responsible for data acquisition, analysis, and interpretation. LBS and PJ drafted the manuscript and DS and JM revised it critically for important intellectual content. All authors gave approval of the final version and agreement to be accountable for all aspects of the work in ensuring that questions related to the accuracy or integrity of any part of the work were appropriately investigated and resolved.

## Funding

This research was supported by National funding from the Portuguese Foundation for Science and Technology within the R&D unit 472, CIPER, (UID/DTP/00447/2013). DS and JM are supported by a scholarship from the Portuguese Foundation for Science and Technology (DS grant: SFRH/BPD/92462/2013; JM grant: SFRH/BD/85742/2012).

### Conflict of interest statement

The authors declare that the research was conducted in the absence of any commercial or financial relationships that could be construed as a potential conflict of interest.

## Abbreviations

BMI: Body mass index
BST: breaks in sedentary time
CRF: cardiorespiratory fitness
HbA1c: Glycated hemoglobin
HOMA-IR: homeostatic model assessment
iAUC: incremental area under the curve
LIPA: low-intensity physical activity
MVPA: moderate-to-vigorous physical activity
T2D: type-2 diabetes.
